# Ineffectiveness of pioglitazone in cognitive impairment induced by cyclophosphamide, methotrexate, and fluorouracil via oxidative stress and neuroinflammation

**DOI:** 10.3389/fnins.2025.1648041

**Published:** 2025-09-04

**Authors:** Ahmad Hamad Alhowail

**Affiliations:** Department of Pharmacology and Toxicology, College of Pharmacy, Qassim University, Buraidah, Saudi Arabia

**Keywords:** chemotherapy, pioglitazone, cognitive dysfunction, oxidative stress, inflammation

## Abstract

**Research objective:**

Chemotherapy is frequently linked to enduring cognitive impairments in individuals who have survived cancer. The cyclophosphamide, methotrexate, and fluorouracil (CMF) regimen is a standard protocol in cancer treatment. Pioglitazone (PGZ), an oral medication used to treat diabetes, has demonstrated neuroprotective effects against certain chemotherapeutic agents, such as doxorubicin. This study aimed to evaluate the efficacy of PGZ in mitigating cognitive dysfunction caused by CMF.

**Materials and methods:**

Forty male rats were allocated into four distinct groups: control, CMF-treated, PGZ-treated, and CMF + PGZ-treated, to evaluate survival rates, body weights, and cognitive performance using the Y-maze, novel object recognition test (NORT), and fear conditioning memory assessments. Furthermore, the investigation included an analysis of mitochondrial complex I activity, reactive oxygen species (ROS), tumor necrosis factor-α (TNF-α), and interleukin-1β (IL-1β) within the hippocampus.

**Results:**

The CMF and CMF + PGZ groups exhibited decreased survival rates (50 and 40%, respectively) and reductions in body weight (16 and 11%, respectively). The Y-maze showed fewer entries and less time in the novel arm, but total entries were unchanged. The NORT revealed less exploration of the novel object in both CMF and CMF + PGZ groups. In fear conditioning, both groups showed reduced freezing time versus control, indicating memory impairment. Furthermore, mitochondrial complex I activity was diminished, and levels of ROS, TNF-α, and IL-1β were elevated in CMF; however, co-treatment with PGZ did not ameliorate these alterations.

**Conclusion:**

The CMF treatment resulted in cognitive dysfunction, and the addition of PGZ did not alleviate this neurotoxicity.

## Graphical abstract

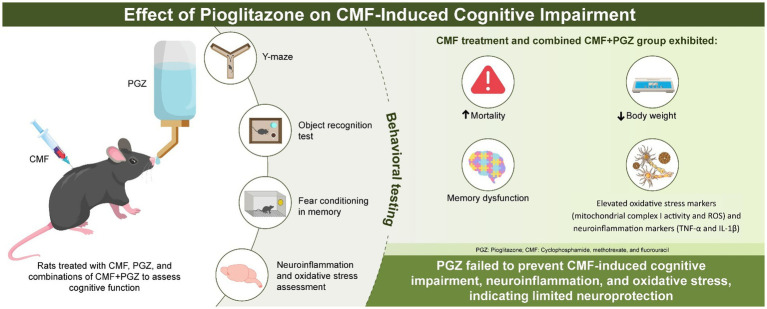

## Introduction

Chemotherapy is a key method for treating cancer and is effective against various tumor types (Anand et al., 2023). However, it also affects healthy tissues owing to its non-selective nature (Blagosklonny, 2023). Additionally, chemotherapy can lead to cellular injury that leads to oxidative stress and inflammation, resulting in toxicity and various side effects (Jiang et al., 2023). An important, yet unexplored side effect of chemotherapy is chemobrain or chemofog, which involves difficulties in concentration, memory, decision-making, learning, and language during and after chemotherapy (Fleming et al., 2023). Up to 70% of cancer survivors experience chemobrain disease, and in 35% of these cases, the symptoms can last for as long as 5 years (Ren et al., 2019). Multiple regions in the brain, including the hippocampus, are primarily responsible for cognitive function and memory formation (Kolibius et al., 2025). Therefore, changes in protein expression or function in the brain can lead to memory impairment (Ding et al., 2023). Currently, the exact causes and mechanisms of chemobrain remain poorly understood.

A regimen of cyclophosphamide, methotrexate, and fluorouracil (CMF) is frequently used to treat different types of cancers, including breast cancer (Park et al., 2017). Cyclophosphamide, an alkylating agent with multiple functions, functions by alkylating DNA, thereby hindering DNA transcription and RNA translation (Ahlmann and Hempel, 2016). Methotrexate acts as a folate antagonist by blocking several enzymes involved in nucleotide synthesis, including dihydrofolate reductase, which facilitates the conversion of dihydrofolate to tetrahydrofolate (Cronstein and Aune, 2020). Fluorouracil, an antimetabolite of the natural nucleobase uracil, inhibits thymidylate synthase, thereby obstructing DNA and RNA syntheses (Sethy and Kundu, 2021). CMF can cross the blood–brain barrier, offering therapeutic effects against cancer cells in the brain (Corley and Allen, 2021). Koppelmans et al. (2012) examined the neuropsychological performance of breast cancer survivors over two decades after receiving adjuvant CMF chemotherapy. Their findings indicated that these individuals scored lower on neuropsychological tests than the random population controls (Koppelmans et al., 2012). Furthermore, studies have shown that CMF administration reduces hippocampal neurogenesis in rodent models of chemobrain, resulting in impaired cognitive function (Briones and Woods, 2011).

Pioglitazone (PGZ), a derivative of thiazolidinedione, serves as an activator of the peroxisome proliferator-activated receptor (PPAR) (Devchand et al., 2018). It has been sanctioned for use in managing diabetes mellitus linked to insulin resistance (Alam et al., 2019). By activating PPAR agonists, PGZ enhances insulin sensitivity, facilitates glucose uptake, and optimizes lipid metabolism (Alam et al., 2019). Moreover, PGZ has shown potential in treating neurodegenerative diseases, highlighting its promise as a therapeutic option for these conditions (Alhowail A. et al., 2022). A previous study has indicated that PGZ positively affects cognitive impairment by stimulating the cholinergic system and reducing neuroinflammation in rat models of neurodegeneration and chemotherapy (Alsaud et al., 2023; Zhao et al., 2021). Pioglitazone (PGZ) has been demonstrated to mitigate diabetes-associated cognitive impairment in rodent models, primarily through the attenuation of neuroinflammatory processes, as evidenced by reduced expression of pro-inflammatory cytokines such as TNF-α, IL-6, and IL-1β, along with improved insulin sensitivity (Saunders et al., 2021). Furthermore, recent preclinical investigations have evaluated the synergistic neuroprotective potential of PGZ in combination with doxorubicin in animal models (Alsaud et al., 2023). The underlying mechanisms proposed for these effects include the enhancement of mitochondrial bioenergetics, suppression of oxidative stress, downregulation of neuroinflammatory signaling pathways, and inhibition of apoptotic cascades (Alsaud et al., 2023; Alhowail, 2024).

The primary aim of this study was to evaluate the effects of CMF on memory function and explore the possibility of using PGZ in conjunction with CMF to mitigate any cognitive impairments induced by CMF.

## Materials and methods

### Chemicals

Cyclophosphamide was sourced from Baxter, located in Mumbai, Maharashtra, India. Methotrexate was sourced from Hospira UK Ltd., based in Leeds, UK. Fluorouracil was procured from United Pharm. Inc., in Korea, situated in Seoul, South Korea. PGZ hydrochloride was obtained from Tabuk Pharmaceuticals in Tabuk, Saudi Arabia. Additionally, Pioglitazone hydrochloride was also sourced from Tabuk Pharmaceutical Manufacturing Co., in Tabuk, Saudi Arabia.

### Animals

Male rats, aged 12 weeks and weighing between 170 and 215 grams, were individually housed in a controlled, pathogen-free environment at the animal facility of Qassim University’s College of Pharmacy. The rats were maintained on a 12-h light/dark cycle, with lights turning on at 6:00 am. They had unrestricted access to water and were provided with a standard chow diet. Daily monitoring was conducted to observe any potential changes. Mortality and body weight were recorded every other day. Behavioral assessments were performed during the light phase. The rats were allocated into four groups, each comprising 10 animals, resulting in a total of 40 rats.

### Drug administration

Rodents received three saline doses intraperitoneally (administered every 3 days) and three cycles of CMF (cyclophosphamide 50 mg/kg, methotrexate 2 mg/kg, and fluorouracil 50 mg/kg). Pioglitazone was administered through drinking water daily at a concentration of 2 mg/mL to achieve an approximate dose of 40 mg/kg/day. Therefore, to ensure each rat received the intended 40 mg/kg/day of PGZ, daily water intake was monitored, with a threshold of 6 mL per day, to maintain consistent dosing (Alsaud et al., 2023; Alhowail, 2024; Rafati et al., 2015). Subsequently, behavioral testing was conducted 1 day after the final dose (Alhowail and Aldubayan, 2023a).

### Y-maze

The Y-maze is a fundamental behavioral test predominantly utilized in rodent research to assess spatial memory and exploratory behavior. The apparatus consists of three arms arranged in a Y-shape (50 × 10 × 15 cm), each typically set at 120° angles from one another. The maze is painted brown to enhance the visibility of the animals. Consistent illumination is provided by overhead lighting. The animals undergo a habituation period in the entire Y-maze 24 h before the test. The study is divided into training and testing sessions. Prior to the training session, visual markers (circles, triangles, and X’s) are placed at the ends of the arms, and one arm is blocked to serve as a novel arm during the testing session. In the 15-min training session, the rats are allowed to freely explore two arms (the start arm and an adjacent arm chosen at random). After a 3-h interval, a 5-min acclimation period in the start arm precedes the testing session, where the rats can explore the entire maze, including the novel arm. The testing session is recorded on video, and data on entries and time spent in the novel arm are collected and analyzed. An arm entry is defined as the entry of more than half of the rat’s body into the arm (Alsikhan et al., 2023).

### Novel object recognition test (NORT)

The Novel Object Recognition (NORT) test is a prevalent behavioral assay in neuroscience, designed to evaluate cognitive function by assessing recognition memory, which includes both learning and memory retention. This test leverages the natural inclination of rodents to explore unfamiliar objects more thoroughly than those they recognize. The experimental setup involves a wooden box with dimensions of 41 × 41 × 41 cm. Initially, the rodents are given a 24-h period to acclimate to this environment before the test begins. During the Familiarization phase, the subjects are presented with two identical objects (teacups) for a 15-min period. Following this, the animals are removed from the environment for a three-hour interval. In the subsequent Retention phase, one of the original objects is replaced with a rectangular box of comparable size, and the animals are allowed to explore for 5 min. The duration of exploration is recorded on video, quantified, and subjected to analysis for further insights (Alsaud et al., 2023).

### Fear conditioning in memory

Fear conditioning is a classical conditioning paradigm extensively employed in neuroscience to investigate associative learning and memory, with a particular emphasis on contextual memory associated with fear. This paradigm serves as a robust and well-characterized model for examining the neural circuits and molecular mechanisms underlying memory formation, storage, and retrieval. The process relies on the interaction between the hippocampal and amygdala circuits. The experiment is structured into two primary phases: acquisition (training) and testing (recall). During the acquisition phase, rats are placed in a standard operant chamber where a stimulus (tone) is paired with an aversive stimulus (electrical foot shock). The animals learn to associate the context (tone) with the electrical shock. In the recall phase, the animals are returned to the same environment to evaluate memory with the tone, but without the electrical shock, by observing freezing behavior (absence of all movement except for respiration). On day 11, the rats undergo a 30-min context habituation without any stimulation. On day 12, the rats are exposed to a tone paired with multiple foot shocks over a duration of 180 s. After a 3-h interval, the rats are returned, and the tone is applied for a shock-free baseline assessment. Fear memory is assessed by measuring the freezing response (immobility, except for respiration) and comparing freezing durations between the treated and control groups (Alhowail and Aldubayan, 2023b).

### Preparation of hippocampal samples

The rodents were humanely euthanized through CO_2_ inhalation, followed by decapitation to facilitate brain extraction. The brain was then rinsed with chilled PBS to remove any blood. Following this, the brain was dissected to isolate the hippocampus, which was subsequently homogenized using a Qsonica homogenizer (30 Hz, Newtown, CT, United States) in conjunction with N-PER lysis buffer (Thermo Scientific, Madison, WI, United States). The resulting homogenate underwent centrifugation at 4°C for 10 min at 12,000 × *g*. The supernatant was then carefully transferred to new Eppendorf tubes, and the protein concentration was quantified using a bicinchoninic acid (BCA) assay, in preparation for enzyme-linked immunosorbent assay (ELISA) analysis.

### ELISA

Upon the preparation of hippocampal samples from both control and treated groups (CMF, PGZ, and CMF + PGZ), the concentrations of TNF-α, IL-1β, and ROS were quantified using ELISA kits. These kits, procured from ABclonal Technology, Woburn, MA, United States, are identified by the following catalog numbers: TNF-α (cat. no. RK00029), IL-1β (cat. no. RK00009), and ROS (cat. no. RK15281). The assays were conducted in accordance with the manufacturers’ protocols. Optical density readings were taken at 450 nm using the ELx800 Microplate Reader from BioTek Instruments, Winooski, VT, United States. The resulting color intensity was compared against standard and control samples to determine the levels of TNF-α, IL-1β, and ROS. Statistical analyses were subsequently performed to interpret the data.

### Mitochondrial complex I activity

The hippocampi were homogenized in phosphate-buffered saline (PBS) and lysis buffer (N-PER™), followed by centrifugation at 12,000 × g at 4°*C* for 10 min. The supernatant was subsequently collected and stored at −80°C prior to analysis. Protein content in each sample was quantified using the Bradford method. Mitochondrial complex I activity was assessed spectrophotometrically at 340 nm, utilizing NADH as the substrate, and was calculated as NADH oxidized per milligram of protein (Shalgum et al., 2019).

### Data analysis

The results are presented as standard error of the mean (SEM). Data were analyzed using GraphPad Prism 10 software. One-way analysis of variance (ANOVA) was followed by Tukey’s *post hoc* test for data analysis. A *p*-value of < 0.05 was considered indicative of statistical significance.

## Results

### Effect of CMF and PGZ treatments on survival rate

**Figure 1:**
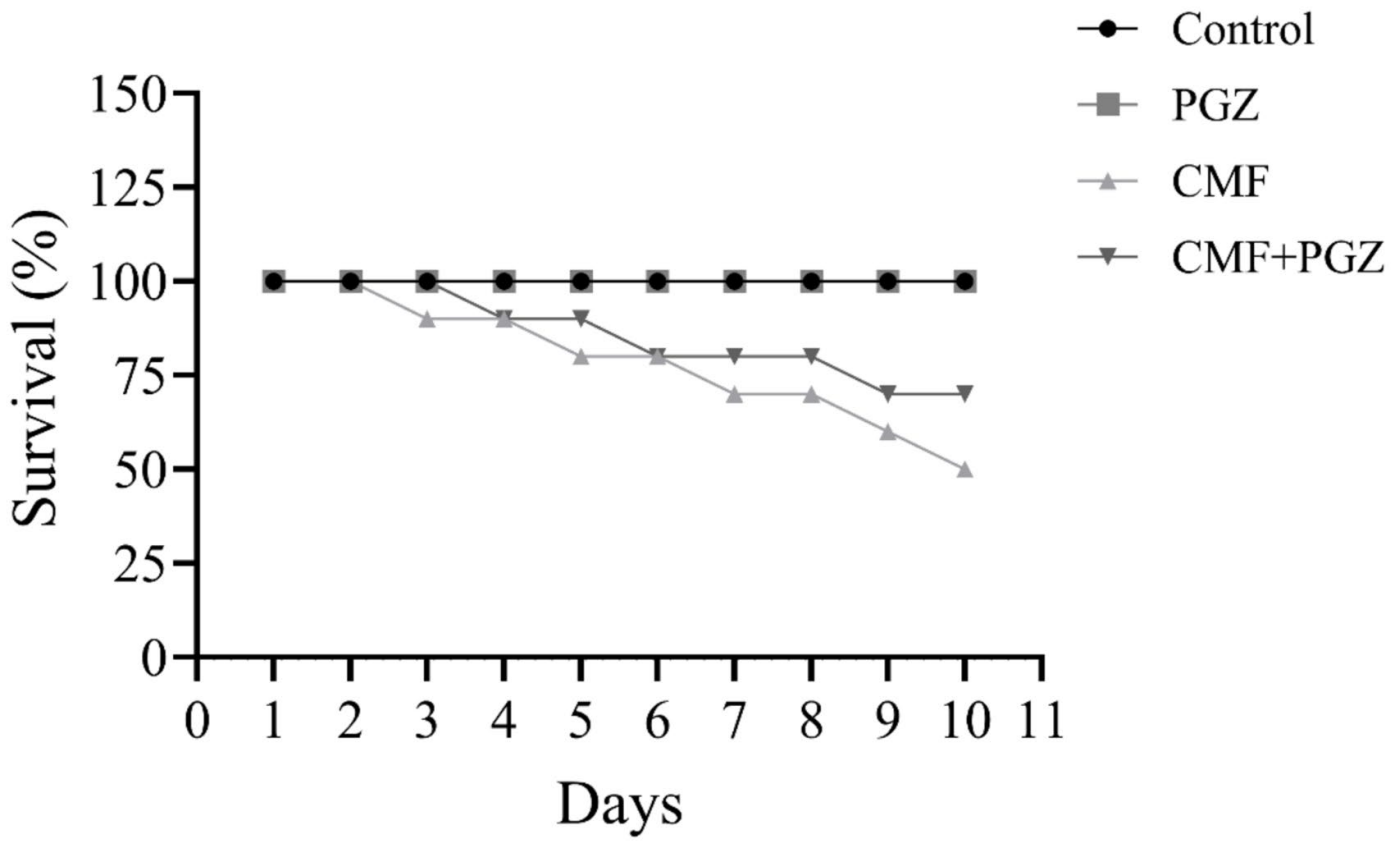
Effects of CMF and PGZ on survival. The survival rate of the rats was determined after 10 days of treatment with CMF, PGZ, CMF + PGZ, and the control. Data represent mean ± SEM (*n* = 10 for each group). CMF, a regimen combining cyclophosphamide, methotrexate, and fluorouracil; PGZ, Pioglitazone.

The CMF treatment affected the survival rates, with a 50% mortality rate observed in the CMF-treated rat cohort. Conversely, the CMF + PGZ group exhibited a 40% mortality rate following the 10-d treatment period (Figure 1).

### Effect of PGZ treatment on CMF-induced loss in body weight

**Figure 2:**
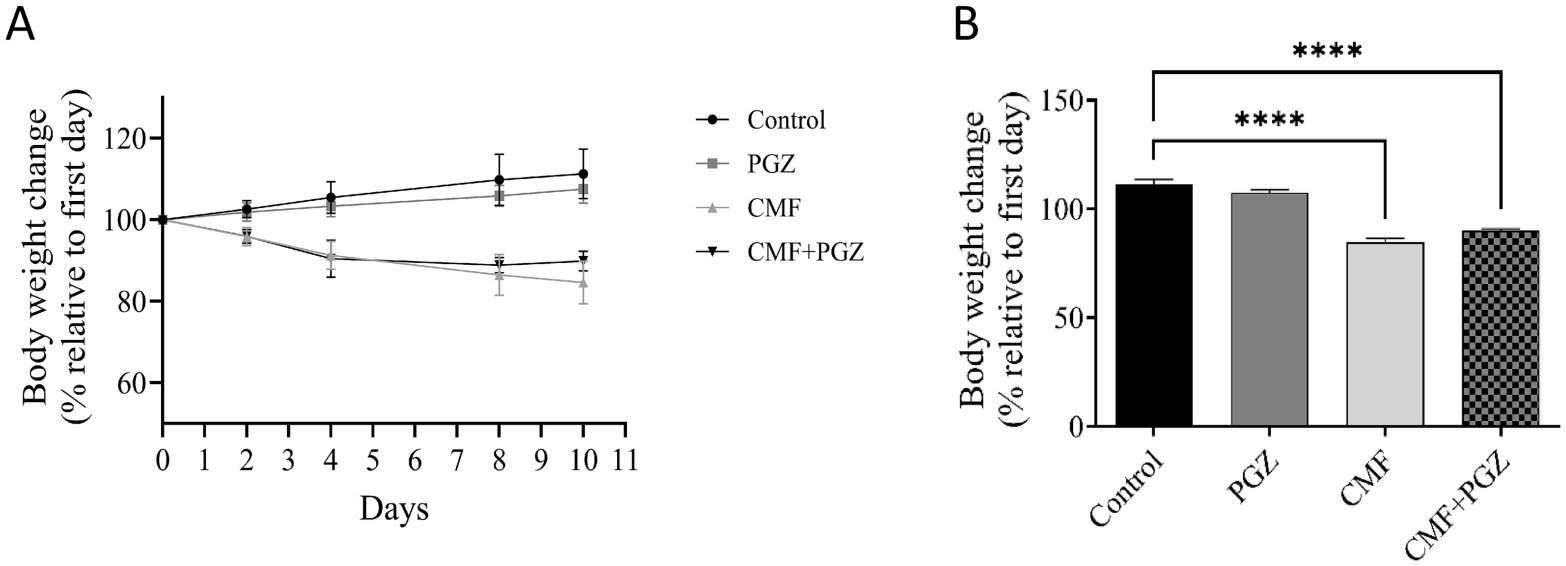
Effects of CMF and PGZ on body weight. **(A)** The body weights of the rats were determined during the study period, before and after treatment with CMF, PGZ, and CMF + PGZ, and the control. **(B)** The body weight difference on the last day of treatment relative to the first day of treatment. Data represent mean ± SEM. CMF, a regimen combining cyclophosphamide, methotrexate, and fluorouracil; PGZ, Pioglitazone. ****p < 0.0001.

The CMF and CMF + PGZ treatments resulted in a gradual reduction in body weight throughout the 10-day study period (approximately 16 and 11%, respectively). In contrast, the control and PGZ alone revealed an increase in body weight (approximately 11 and 7%, respectively) (Figures 2A,B).

### Effect of PGZ treatment on CMF-induced cognitive function impairment

**Figure 3:**
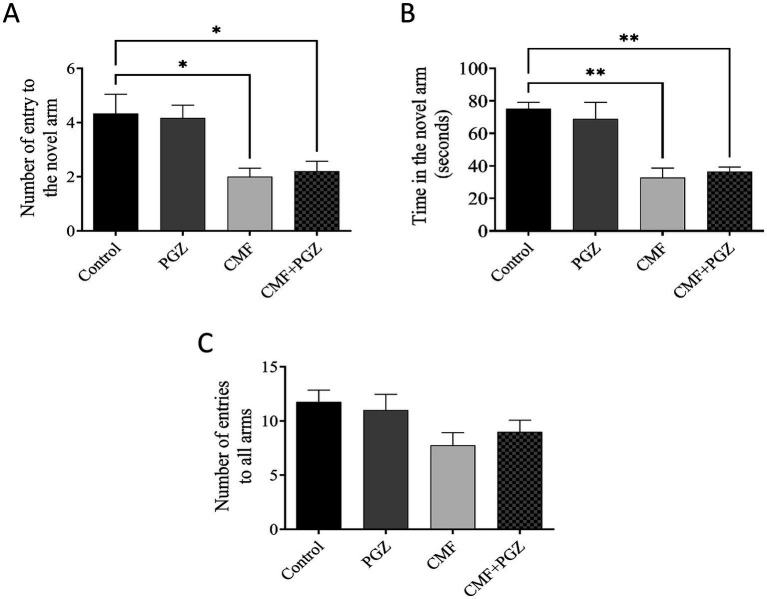
Behavioral tests on rats using the Y-maze test. Following a 10-day regimen involving CMF, PGZ, and their combination (CMF + PGZ), the Y-maze test was administered. **(A)** The analysis focused on the influence of CMF and PGZ on the frequency of entries into the novel arm. **(B)** The study examined the impact of CMF and PGZ on the duration spent in the novel arm. **(C)** The research investigated the effects of CMF and PGZ on the number of entries into all novel arms of the Y-maze. CMF, a regimen combining cyclophosphamide, methotrexate, and fluorouracil; PGZ, Pioglitazone; NORT, object recognition test. * *p* < 0.05 and ** *p* < 0.01.

In the Y-maze assessment, the groups treated with CMF and CMF + PGZ demonstrated a marked decrease in both the number of entries and the duration spent in the novel arm when compared to the control group. Importantly, a substantial difference was observed in these parameters between the PGZ groups (Figures 3A,B). These observations indicate that CMF treatment impairs the ability to form new memories after therapy. To evaluate any potential lethargy in the subjects, the total number of entries into all arms was recorded, showing no significant differences (Figure 3C).

### Effect of PGZ treatment on the CMF novel object recognition test

**Figure 4:**
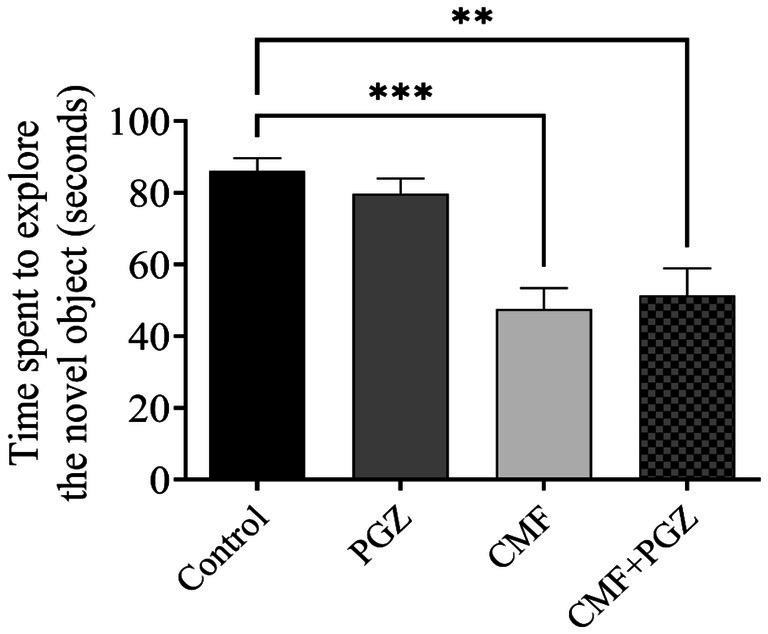
Behavioral tests on rats using the novel object recognition test (NORT). The NORT was performed after the 10-d treatment with CMF, PGZ, and CMF + PGZ to assess the exploration time in NORT. CMF, a regimen combining cyclophosphamide, methotrexate, and fluorouracil; PGZ, Pioglitazone; NORT, object recognition test. **p < 0.01 and ***p < 0.001.

Figure 4 illustrates a decrease in exploration time of the novel object in both the CMF and CMF + PGZ groups compared to the control group, while no significant difference is observed between the PGZ group and the control group.

### Effect of PGZ treatment on the CMF fear conditioning memory

**Figure 5:**
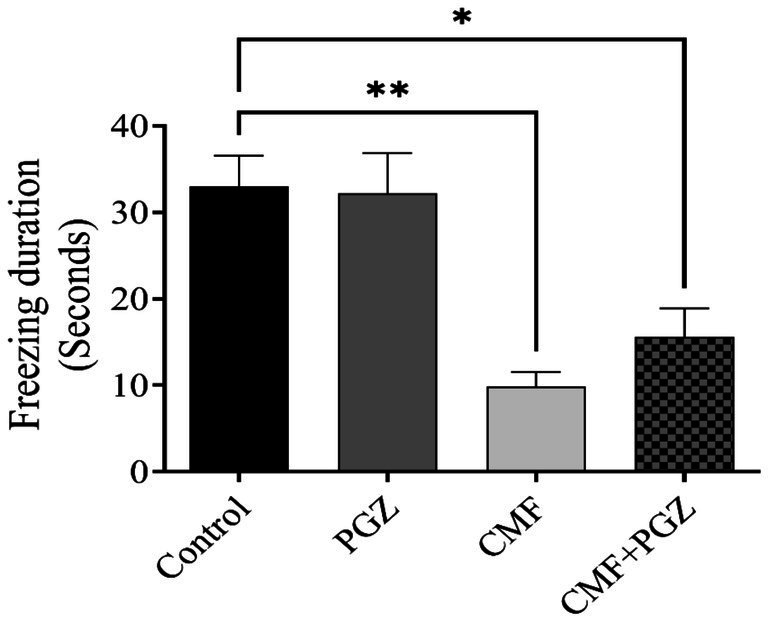
Behavioral tests on rats using fear conditioning memory. The fear conditioning memory test was performed after a 10-day treatment with CMF, PGZ, and CMF + PGZ to assess freezing time in fear conditioning memory tasks. CMF, a regimen combining cyclophosphamide, methotrexate, and fluorouracil; PGZ, Pioglitazone; NORT, object recognition test. *p < 0.05 and **p < 0.01.

Figure 5 shows the fear conditioning memory results, which indicate a reduction in freezing time in the CMF and CMF + PGZ groups compared to the control. In contrast, there was no PGZ in contrast to the control.

### Effect of PGZ treatment on CMF-induced oxidative stress

**Figure 6:**
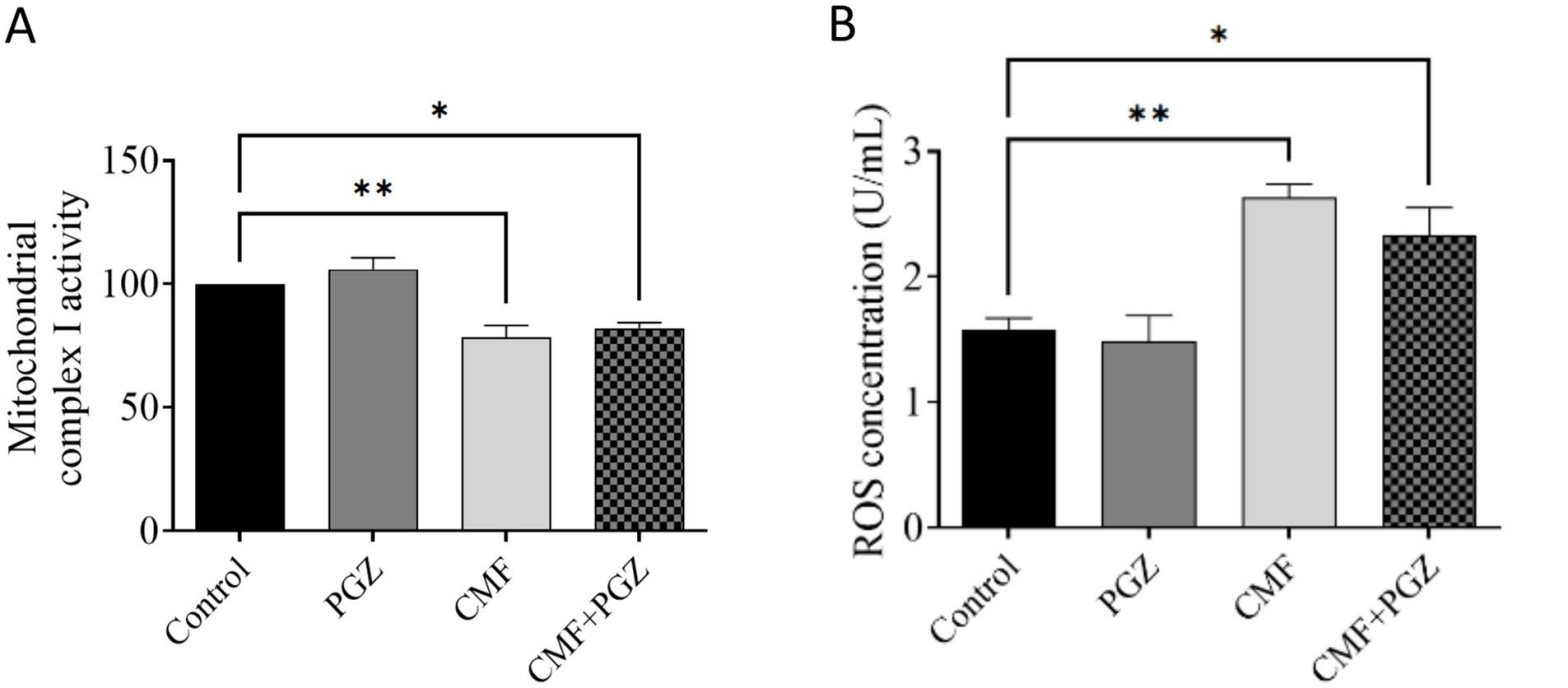
Effects of CMF and PGZ on mitochondrial complex I activity and ROS levels in the rat brain. **(A)** Analysis revealed a reduction in mitochondrial complex I activity and **(B)** elevated ROS levels in rat brains following a 10-day treatment regimen of CMF and CMF+PGZ (the control group did not receive any treatment). Data represent the mean ± SEM. *p < 0.05 and **p < 0.01. CMF, a regimen combining cyclophosphamide, methotrexate, and fluorouracil; PGZ, Pioglitazone; ROS, reactive oxygen species.

The analysis demonstrated a significant decrease in mitochondrial complex I activity and increased ROS expression levels in both the CMF and CMF + PGZ treatment groups, relative to the control. Protein levels were normalized to the total protein content and expressed as a percentage of the control group (set to 100%) (Figures 6A,B).

### Effect of PGZ treatment on CMF-induced neuroinflammation

**Figure 7:**
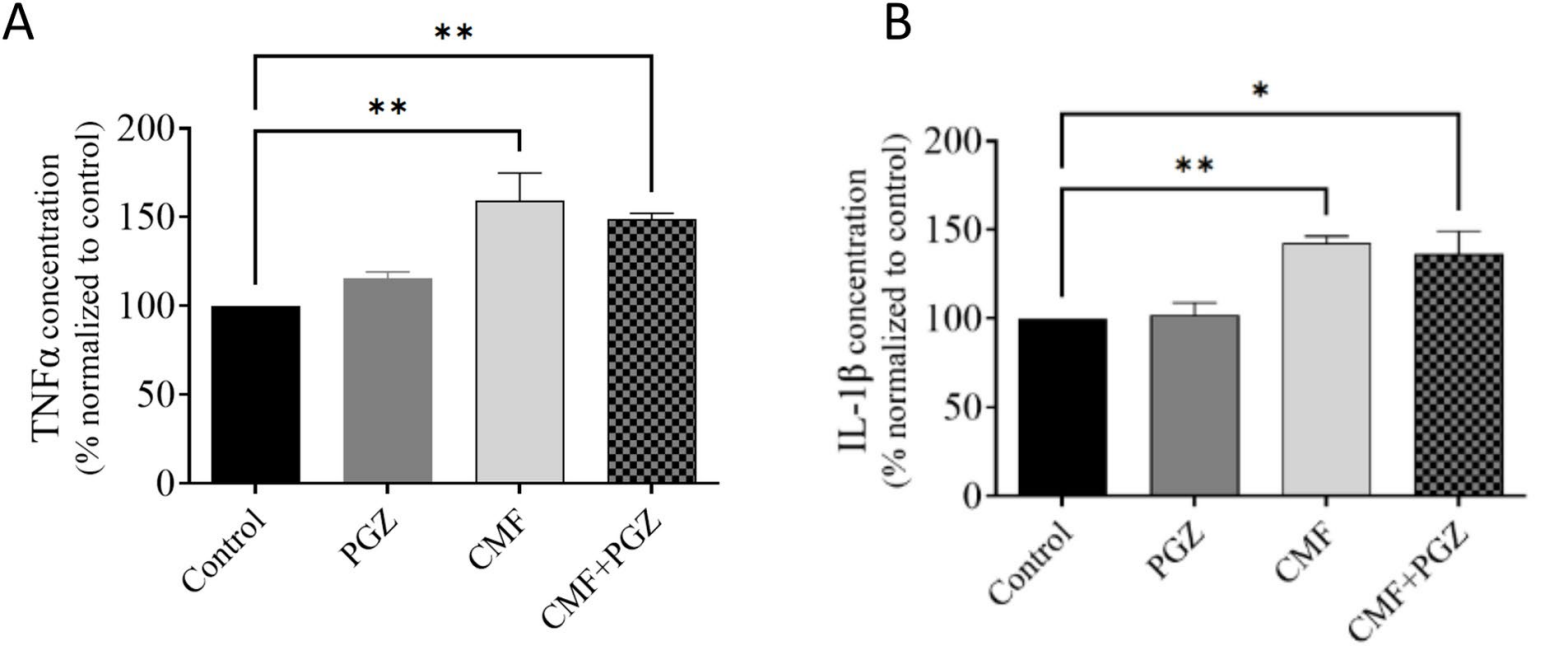
Effects of CMF and PGZ on TNF-α and IL-1β expression in the rat brain. The effects of CMF and PGZ on **(A)** TNF-α and **(B)** IL-1β levels in the rat brain were evaluated by ELISA after 10 days of treatment with CMF, PGZ, and CMF+PGZ (the control group did not receive any treatment). Data represent the mean ± SEM. *p < 0.05 and **p < 0.01. CMF, a regimen combining cyclophosphamide, methotrexate, and fluorouracil; PGZ, Pioglitazone; TNF-α, tumor necrosis factor-α; and IL-1β, interleukin-1β.

Results analysis demonstrated significantly increased TNF-α and IL-1β expression in both CMF and CMF + PGZ treatment groups, relative to the control; however, the levels of these proteins were comparably elevated in the CMF + PGZ group. Protein levels were normalized to the total protein content and expressed as a percentage of the control group (set at 100%) (Figures 7A,B).

## Discussion

This research explored the influence of the CMF chemotherapy regimen on memory function in rats by employing behavioral assessments such as the Y-maze test, the Novel Object Recognition Test (NORT), and fear conditioning memory tasks. In chemotherapy protocols, it is common to combine multiple chemotherapeutic agents to reduce the possibility of drug resistance and to improve therapeutic outcomes (Anand et al., 2023). Both clinical and experimental studies have previously identified memory impairments as a consequence of chemotherapy (Alhowail and Aldubayan, 2023b). Our previous study in rats demonstrated that the CMF regimen impaired memory, confirming a correlation between the CMF regimen and cognitive dysfunction (Alhowail A. H. et al., 2022). In addition, our previous findings have shown that PGZ has promise in ameliorating DOX-induced neurodegeneration and chemobrain damage, as well as in diabetic and Alzheimer’s disease (Alsaud et al., 2023; Alhowail, 2024; Gao et al., 2017; Seok et al., 2019). Therefore, we hypothesized that PGZ could exert a protective effect against CMF-induced cognitive dysfunction, as assessed using the Y-maze, NORT, and fear conditioning memory tests.

Our data indicated significant toxicity associated with the CMF regimen. In particular, the survival rate decreased by 40% in the CMF group, while the combination of CMF and PGZ reduced the survival rate by 30%. Therefore, this is considered a slight mitigation of CMF toxicity by PGZ. These results raised serious concerns regarding the safety and efficacy of PGZ in reducing CMF toxicity. Additionally, body weight data on the safety of PGZ in relation to CMF toxicity were reviewed. These findings indicate a significant 16% reduction in body weight following CMF treatment. However, the addition of PGZ slightly mitigated this weight reduction; the reduction was 11%. Therefore, it is important to understand the mechanisms underlying this effect. In contrast, body weight increased by approximately 11% in the control group and 7% in the PGZ group, respectively.

Cognitive function was assessed using the Y-maze test, NORT, and fear conditioning memory tasks. These methods evaluate spatial recognition and associative learning. The Y-maze test quantifies exploratory behavior in novel environments, whereas the NORT assesses the recognition memory of novel objects (Kim et al., 2023; Kirby et al., 2018). Fear conditioning assesses the formation of associations between neutral and aversive stimuli (Alhowail and Aldubayan, 2023b). These behavioral paradigms provide a comprehensive assessment of various cognitive domains. Analysis of spatial memory using the Y-maze revealed a significant decrease in arm entries and the time spent in the novel arm in the CMF-treated group. While PGZ co-treatment did not improve cognitive impairment. Similarly, NORT results demonstrated reduced novel object exploration in the CMF-treated group, compared with controls. Moreover, the combination of the CMF and PGZ failed to improve novel object exploration. These data suggest that the CMF treatment may negatively affect exploratory behavior. Moreover, while fear conditioning memory tasks are dependent on the hippocampus, they originate in the amygdala (Kim and Cho, 2020). Our results diverged from previous findings, indicating a statistically significant reduction in freezing time in the CMF group (Alhowail A. H. et al., 2022). However, the addition of PGZ co-treatment did not yield further improvements in freezing time. The CMF-treated groups exhibited shorter freezing times than the control group. Collectively, these findings demonstrate the ineffectiveness of PGZ in mitigating CMF-induced toxicity.

We administered a CMF treatment to introduce cognitive impairment known to occur after this chemotherapy treatment. This study is the first to use CMF medications to predict induced cognitive impairment in combination with pioglitazone. Previous findings revealed that CMF reduces neurogenesis and increases neuroinflammation, inducing cognitive dysfunction (Alhowai et al., 2022; Murillo et al., 2023). Previous studies also revealed that pioglitazone improves neuronal function and reduces neuroinflammation in neurodegenerative diseases (Alzheimer’s and Parkinson’s) as well as diabetes (Saunders et al., 2021; Zamanian et al., 2023). Therefore, we hypothesized that pioglitazone could improve neuronal circuits, reflected in behavioral function. Unfortunately, after conducting the experiment, we did not see much improvement. However, there was a slight mitigation of CMF toxicity, shown by improved survival rate (30% mortality versus 40% in CMF only) and reduced weight loss (11% versus 16%). Furthermore, behavioral assessment results showed no effect in the Y-maze by combining pioglitazone with CMF, whereas slight mitigation was detected in the NOR and fear conditioning memory tests. Therefore, pioglitazone was unable to reverse the effects on neurons after CMF treatment. Our interpretation of these results is that CMF may cause irreversible damage to neurons. Further study is required to investigate the mechanisms of CMF-induced cognitive impairment to better understand its cellular effects and allow for the development of neurotherapies.

Chemotherapy is recognized for inducing oxidative stress, which is characterized by compromised mitochondrial function and elevated levels of reactive oxygen species (ROS) (Zhang et al., 2019). Chemotherapeutic agents such as doxorubicin and cisplatin primarily exert cytotoxic effects by increasing intracellular ROS (Ongnok et al., 2020). Similarly, cyclophosphamide, methotrexate, and fluorouracil (CMF) can promote ROS generation either directly or by impairing mitochondrial function (Sahu et al., 2021). In the present study, CMF treatment significantly impaired mitochondrial complex I activity and elevated ROS levels, indicating the induction of oxidative stress. However, co-treatment with pioglitazone (PGZ) did not mitigate the mitochondrial dysfunction, as evidenced by reducing complex I activity by reducing the oxidized NADH to NAD^+^ that is essential for energy production by formation of adenosine triphosphate (ATP), and failed to rescue the increase in ROS levels compared to the control group, suggesting that PGZ was ineffective in reducing CMF-induced oxidative stress.

Elevated concentrations of pro-inflammatory cytokines such as TNF-α and IL-1β in the brain are indicative of neuroinflammation, a pathological condition that can impair mitochondrial function and trigger oxidative stress (Bhol et al., 2024). In this study, CMF treatment modestly increased levels of TNF-α and IL-1β, consistent with the presence of neuroinflammation. Concurrently, impaired mitochondrial function and elevated ROS levels further supported the occurrence of oxidative stress. These pathological changes are believed to contribute to the cognitive deficits observed following CMF therapy. Although this study hypothesized that PGZ could alleviate CMF-induced cognitive impairment by reducing oxidative damage and neuroinflammation, the results showed that co-treatment with PGZ was ineffective in attenuating the oxidative stress and neuroinflammatory response.

There are a number of strengths and limitations associated with this study. The purpose of this study is to investigate the effects of CMF on cognitive impairment. Additionally, this is the first study to investigate the combination of PGZ as a potential protective benefit with CMF. The use of a homogenous rat population (including rats of the same strain, age, and weight, as well as those that have been shown to be cancer-free) to isolate the impact of CMF and so minimize confounding variables is one of the strengths of this study. Additionally, to ensure consistency with clinical practice, the researchers used medications prescribed by hospitals and obtained from commercial sources. Moreover, our laboratory’s current technical and resource constraints have prevented us from conducting thorough histological analyses to corroborate our functional and behavioral findings. Future investigations should include histological examinations of neuronal architecture, cellular integrity, and any potential pathological changes in these brain regions to validate and build upon our results.

## Conclusion

This study investigated the effects of CMF on neurotoxicity and memory dysfunction in rats by assessing survival rates, body weight, and neuronal function. This was achieved using the Y-maze, NORT, and fear conditioning memory behavioral tasks. This study also evaluated the protective potential of PGZ co-administration in mitigating cognitive impairment. Overall, our findings suggest that PGZ was ineffective in mitigating CMF-induced neurotoxicity, as reflected in cognitive function, oxidative stress, and neuroinflammation. Our findings highlight the need for further studies to explore further mechanistic pathways for alleviating chemotherapy-related cognitive impairments in cancer survivors.

## Data Availability

The original contributions presented in the study are included in the article/supplementary material, further inquiries can be directed to the corresponding author.
